# X-chromosome variants are associated with aldosterone producing adenomas

**DOI:** 10.1038/s41598-021-89986-8

**Published:** 2021-05-18

**Authors:** Ravi Kumar Dutta, Malin Larsson, Thomas Arnesen, Anette Heie, Martin Walz, Piero Alesina, Oliver Gimm, Peter Söderkvist

**Affiliations:** 1 Division of Cell Biology, Department of Biomedical and Clinical Sciences, Linköping University, 58183 Linköping, Sweden; 2 Science for Life Laboratory, Department of Physics, Chemistry and Biology, Linköping University, 58183 Linköping, Sweden; 3 Department of Surgery, Haukeland University Hospital, Bergen, Norway; 4 Department of Biological Sciences, University of Bergen, Bergen, Norway; 5 Department of Biosciences, University of Bergen, Bergen, Norway; 6 Klinik Für Chirurgie and Zentrum Für Minimal Invasive Chirurgie, Klinikum Essen-Mitte, Essen, Germany; 7 Department of Surgery and Department of Biomedical and Clinical Sciences, Linköping University, 58183 Linköping, Sweden; 8 Department of Clinical and Experimental Medicine, Linköping University, 58183 Linköping, Sweden

**Keywords:** Genetics, Genetic association study, Genome-wide association studies

## Abstract

Aldosterone-producing adenomas (APAs) are a major cause of primary aldosteronism (PA) and are characterized by constitutively producing aldosterone, which leads to hypertension. Several mutations have been identified in ion channels or ion channel-associated genes that result in APAs. To date, no studies have used a genome-wide association study (GWAS) approach to search for predisposing loci for APAs. Thus, we investigated Scandinavian APA cases (n = 35) and Swedish controls (n = 60) in a GWAS and discovered a susceptibility locus on chromosome Xq13.3 (rs2224095, OR = 7.9, 95% CI = 2.8–22.4, P = 1 × 10^–7^) in a 4-Mb region that was significantly associated with APA. Direct genotyping of sentinel SNP rs2224095 in a replication cohort of APAs (n = 83) and a control group (n = 740) revealed persistently strong significance (OR = 6.1, 95% CI = 3.5–10.6, *p* < 0.0005). We sequenced an adjacent gene, *MAGEE1*, of the sentinel SNP and identified a rare variant in one APA, p.Gly327Glu, which is complementary to other mutations in our primary cohort. Expression quantitative trait loci (eQTL) were investigated on the X-chromosome, and 24 trans-eQTL were identified. Some of the genes identified by trans-eQTL point towards a novel mechanistic explanation for the association of the SNPs with APAs. In conclusion, our study provides further insights into the genetic basis of APAs.

## Introduction

According to the World Health Organization (WHO), essential hypertension is one of the major risk factors for cardiovascular disease and death worldwide^1^. Endocrine hypertension (including Conn syndrome) accounts for 5–15% of essential hypertension and is caused by a hormone imbalance, especially involving the pituitary or adrenal gland. Primary aldosteronism (PA) is a subtype of endocrine hypertension in which the adrenal cortex constitutively produces an excess of aldosterone^2,3^. In the majority of PA cases, the excess of aldosterone is produced by adenomas (APAs) or bilateral adrenal hyperplasia^4,5^.

Physiologically, zona glomerulosa (ZG) cells of the adrenal cortex produce aldosterone in response to renin-angiotensin II signaling and increase serum potassium levels^6^. The binding of angiotensin II to its receptor blocks the potassium channels and causes depolarization of the cell membrane. This generates an action potential that opens voltage-gated Ca^2+^ channels, which leads to an influx of Ca^2+^. Likewise, changes in serum K^+^ activate voltage-gated Ca^2+^ channels in ZG cells and enhance the intracellular Ca^2+^concentration, which lead increased aldosterone production. In PA, autonomous production of aldosterone is found to be independent of angiotensin II signaling due to the presence of benign tumors of the adrenal cortex^4,7^. High levels of aldosterone lead to the suppression of plasma renin, high blood pressure, hypokalemia, and a high risk for severe cardiovascular events^8^.

Thus far, somatic mutations in *KCNJ5*, *ATP2B3*, *ATP1A1*, *CACNA1D,* or *CLCN2* have been found in a majority of APAs^2,9–13^. All mutations in these genes result in chronic depolarization of the cell membrane, the opening of voltage-gated Ca^2+^ channels, cellular Ca^2+^ influx, and aldosterone production. Rarely, somatic mutations are also found in the proto-oncogene *CTNNB1* encoding the transcription factor β-catenin. Mutations are mainly found in the phosphorylation site of β-catenin, which leads to the disruption of ubiquinated degradation and the accumulation of β-catenin in nucleus-regulating target genes, such as aldosterone synthase (*CYP11B2*)^14^.

Familial occurrence of PA has been reported and is divided into four forms: familial hyperaldosteronism 1–4 (FH I—FH IV). FH-I occurs by unequal crossing over of the genes *CYP11B1* and *CYP11B2,* and the resulting hybrid gene includes the promoter region of *CYP11B1* and the majority of the coding sequence of *CYP11B2*^15^. Consequently, all three cell layers of the adrenal cortex produce aldosterone regulated by adrenocorticotropic hormone (ACTH) instead of angiotensin II. Regarding FH II–IV, germline mutations in *CLCN2*, *KCNJ5,* and *CACNA1H* have been found in affected individuals^2,16–18^.

Previously, a genome-wide association study (GWAS) was performed on a population of individuals with an elevated aldosterone-to-renin ratio (ARR)^19^. A GWAS locus was identified on chromosome 5q32, but no mutation has been reported from loci associated with APAs. In this study, we performed an SNP array analysis on 35 APA patients and 60 population controls for a genome-wide association (GWA) analysis. A susceptibility locus on the X-chromosome was identified, and genetic variants in the *MAGEE1* gene were found to carry an increased risk for APAs.

## Results

### Identification of susceptibility loci

**Figure 1:**
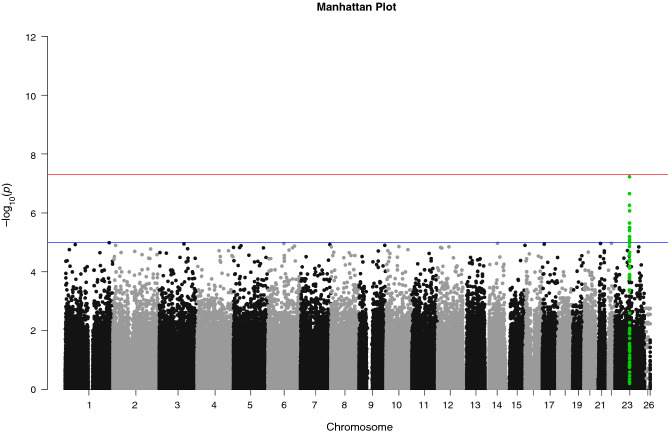
**Manhattan plot.** Genome-wide association results for aldosterone-producing adenomas (APAs). Manhattan plot of *P* values in –log_10_ scale from the logistic regression with sex as a covariate on 727,307 SNPs (from 33 cases and 58 controls). The horizontal lines indicate the genome-wide significance threshold (red line = 1X10^–8^, blue line = 1X10^–6^). Green dots show the 100 most significant SNPs in the region of the susceptibility loci.

**Figure 2:**
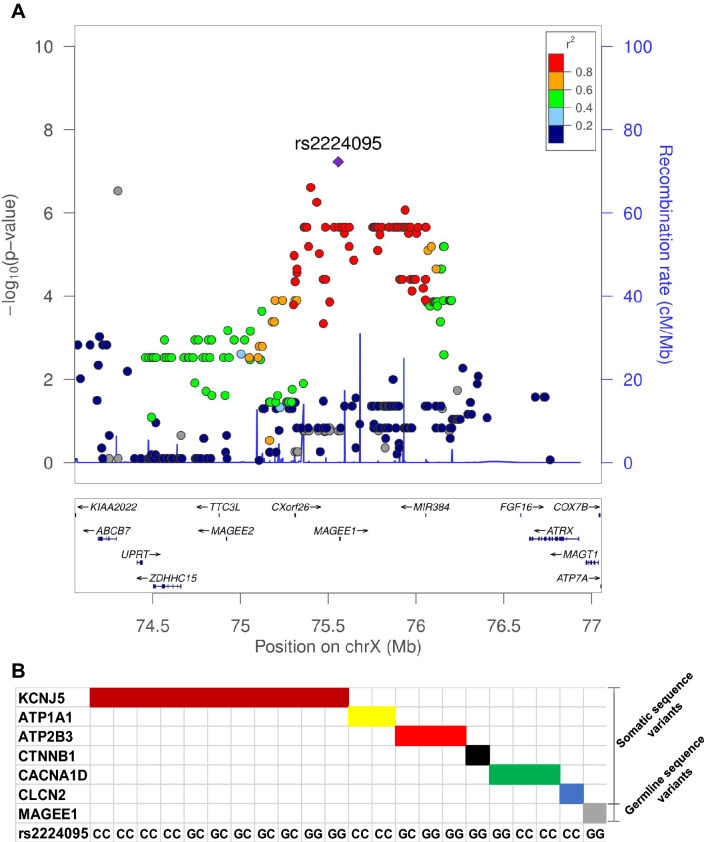
(**A**) **Regional association plot.** The plot shows the association results of SNPs of the GWAS samples and the recombination rates. The –log10 P values (y axis) of the SNPs are shown according to their X-chromosome positions (X axis). The lead SNP (rs2224095) is shown as a diamond. The intensity of each symbol reflects the extent of LD with rs2224095. Genetic recombination rates were estimated using HapMap samples from Utah residents of Western and Northern European ancestry (CEU) and are shown as a blue line. Physical positions are based on hg18. **(B)** A summary of mutations and genotype of sentinel SNP (rs2224095) identified in 24/35 of our cohort of APAs. The remaining APAs had no mutations in the known susceptibility genes.

Genome-wide genotyping was performed on 35 APAs and 60 Swedish controls. The strongest association was found in chromosomal region Xq13.3 (rs2224095 (G/C), OR = 7.9, 95%CI = 2.8–22.4, *P* = 1 × 10^–7^), which is located upstream of the *MAGEE1* gene (Figs. 1 and 2)^20^. We further genotyped an additional cohort of 83 APAs (Norwegian cohort) and 740 healthy Swedish controls for the index SNP rs2224095. The frequencies of the homozygous wild type (control = 547/740, case = 63/118) and homozygous variant for the polymorphism (control = 86, case = 30) of SNP rs2224095 were used for an allelic test. The frequency of the risk allele was higher in patients with APAs than in controls with an odds ratio (OR) of 6.1 (CI 3.5–10.6, *p* < 0.0005).

### Sequencing of MAGEE1

**Figure 3:**
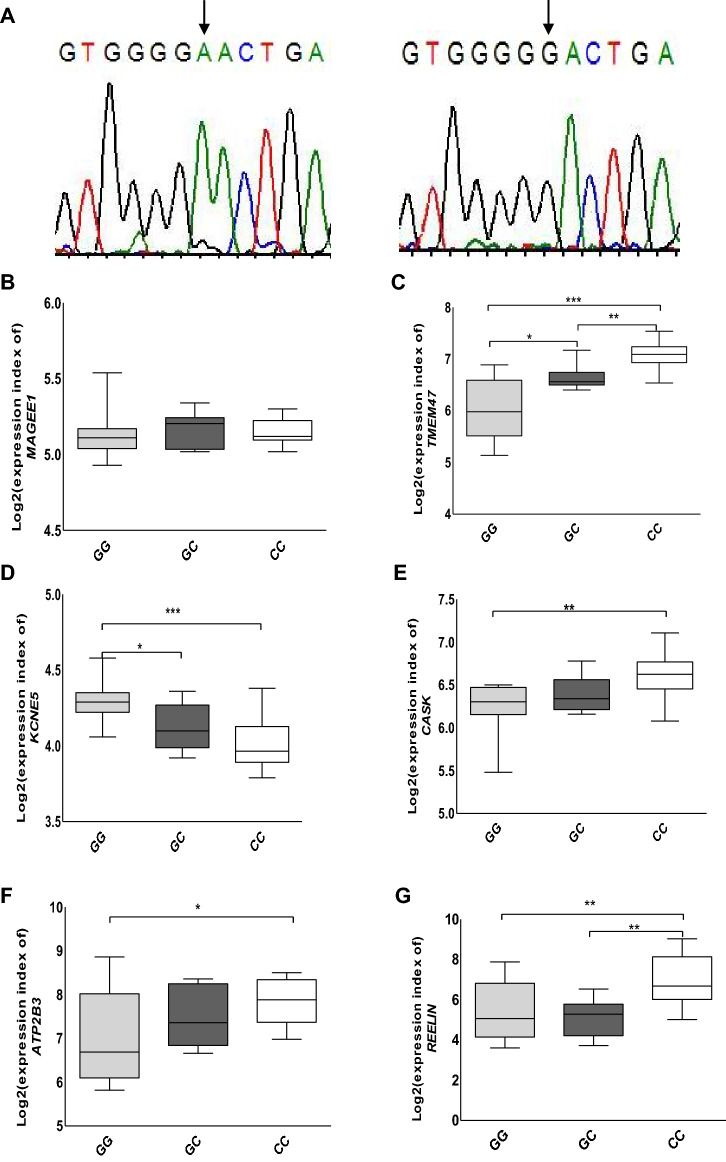
**Sequencing of MAGEE1 and eQTL analysis.** (**A**) The germline sequence variant identified in *MAGEE1* (left) in comparison to the normal sequences from a non-mutated sample (right). (**B-G**) Log2 expression of *MAGEE1*, *TMEM47*, *KCNE5*, *CASK*, *ATP2B3,* and *REELIN* after normalization of mRNA microarray data from patient tissue. *P < 0.05, **P < 0.005, ***P < 0.0005, 2-tailed student t-test.

The sentinel SNP 2,224,095 is located on the 3’ end of the *MAGEE1* gene, which is expressed in APAs (Figs. 2 and 3B). Therefore, we sequenced *MAGEE1* in APAs. We identified a rare heterozygous germline mutation in *MAGEE1* in one case at c.980G > A (rs145140241, p.Gly327Glu) (Table 1) with a minor allele frequency (MAF) of 0.002 on gnomAD (https://gnomad.broadinstitute.org), as well as in the Swedish 1000 genome database (MAF = 0.0005, SweGen, https://swegen-exac.nbis.se/) (Fig. 3A). Polyphen2, SIFT, and Mutation Assessor analysis predicted the single amino acid substitution to be damaging. Patients with the *MAGEE1* mutation had a normal allele (G) for SNP 2,224,095.

**Table 1:** Clinical characteristics of the patient with aldosterone producing adenomas and a germline mutation in *MAGEE1* identified in this study.

Case ID	Gender	Age (years)	Aldosterone (ng/l)	Renin (ng/mL/h)	Tumor size (mm)	Gene	Mutation	Mutation	Protein alteration
B7	M	49.8	1180	0.3	40	*MAGEE1*	Germline	c.980G > A	p.Gly327Glu

### eQTL analysis

To gain further insight into the functional basis of SNP rs2224095, we performed an expression quantitative trait loci (eQTL) analysis using expression data from 34 APAs (unpublished data). The eQTL analysis was performed between the SNP rs2224095 genotype and the expression of genes mapped within ± 1 Mb of SNP rs2224095 for loci (cis-eQTLs) and the entire X-chromosome for distant (trans-eQTL) genes. Sex and age were accounted for as covariates^21^. eQTL analysis is a rigorous method that has a higher threshold for trans-eQTL, which makes it more stringent and potentially biases their detection.

We detected 24 trans-eQTL. The strongest association between the rs2224095 genotype and gene expression was with *TMEM47* (trans-eQTLs), with the risk allele (C) associated with higher expression, which remained significant after adjusting for multiple testing (*P* = 2.7 X 10^–5^, FDR = 0.05) (Fig. 3C, supplementary Table 1). The expression of *KCNE5* (*p* < 0.05) and *CASK* (*p* < 0.05) genes also displayed a strong association with the risk allele (C) (Figs. 3D–E). *TMEM47* and *CASK* revealed higher expression with the risk allele, while *KCNE5* showed significantly lower levels of mRNA expression. We also explored the association of sentinel SNP rs2224095 with genes in the Genotype-Tissue Expression (GTEx) Portal (https://www.gtexportal.org/home/). In the GTEx Portal, rs 2,224,095 was identified as an eQTL for *ZDHHC15* in the adrenal gland. We did not find a significant difference regarding the expression of *ZDHHC15* among the genotypes (Supplementary Fig. 3E). ZDHHC15 is involved in protein palmitoylation. There are no reports that show any connection between *ZDHHC15* expression and APA formation. The coding sequences of TMEM47, KCNE5, CASK, and ZDHHC15 were sequenced in a subset of 14 tumors of the GWAS discovery cohort, but no somatic or germline mutations were detected in these tumors.

A mutation on the X-chromosome gene *ATP2B3* has been reported previously in sporadic APAs at a frequency of approximately 2%^9,22^. However, *ATP2B3* is located 77.12 Mb downstream of the GWAS-identified sentinel SNP. We further examined whether the risk allele of SNP rs2224095 influenced *ATP2B3* expression. *ATP2B3* showed significantly higher levels of mRNA expression associated with the risk allele (Fig. 3F), indicating a potential impact of this SNP on *ATP2B3*. At present, there is no evidence that links the direct connection of genes in the GWAS locus regulating *ATP2B3*.

## Discussion

Sporadic APA is a rare disease, and so far, no GWAS has previously been performed on them. We performed a GWA analysis and discovered one locus on Xq13.3 that is significantly associated with APAs. We detected 29 eQTL by using expression array data from the same cases.

The strongest association was associated with SNP 2,224,095, which is located at the 3’ end of the *MAGEE1* gene. *MAGEE1* belongs to the MAGE family of genes, which is mainly clustered on the X-chromosome and evolutionarily conserved among eukaryotes^23^. Some members of the MAGE family of proteins are involved in ubiquitination. They bind with the ring-like structures of E3 ubiquitin ligases and facilitate binding to the substrate^24^. The *MAGEE1* gene is reported to be mutated in malignant melanoma (7%) and ovarian clear cell carcinoma (19%) (OCCC)^25,26^. Patients with *MAGEE1* mutation in OCCC have the worst overall survival. The expression of MAGEE1 and its mutant has shown an antiproliferative effect in an in-vitro study^25^.

Here, we report a rare variant, c.980G > A (p.Gly327Glu, rs145140241) (Table 1), in the *MAGEE1* gene in one APA that is homozygous for the normal allele (G) of the sentinel SNP rs2224095. The reference amino acid Gly327 is highly conserved among homologous proteins and lies in the MAGE homology domain (MHD), which is involved in binding with E3 ubiquitin ligases^23^. MHD of MAGEE1 is frequently mutated in both melanoma and OCCC and is predicted to be damaging. The APA patient carrying this variant had enormous plasma aldosterone levels (1180 ng/l) and a larger tumor (40-mm diameter) (Table 1), suggesting that a loss of antiproliferative effect of MAGEE1 leads to a larger tumor and produces higher aldosterone. The patient with this variant did not carry mutations in any other of the susceptibility genes identified for APAs thus far. This patient does not have any history of malignant melanoma.

Association studies have demonstrated that affected genes may be located up to several megabases away from the phenotype-associated locus, and the expression of the most adjacent gene is not necessarily altered. We used the eQTL approach to identify genes associated with the sentinel SNP. This approach is a straightforward way to link a non-coding SNP to coding regions (genes). Mapping eQTL-target gene associations in tumor tissue adds additional challenges. Tumors acquire frequent genetic and epigenetic alterations, which can substantially affect gene expression. However, APAs are benign tumors with less genomic heterogeneity and fewer somatic mutations (0.12/Mb) compared to malignant tumors^2^. Since the GWAS locus was located on the X-chromosome, the trans-eQTL analysis was confined to the X chromosome. We found 24 trans-eQTL with FDR < 0.05, and the strongest association was found for the *TMEM47* gene. At present, there are no reports showing any connection between SNP rs2224095 and *TMEM47* expression. Among other eQTL located on the X-chromosome, two candidate genes were identified and may be functionally associated with APA: *CASK* (calcium/calmodulin-dependent serine protein kinase) and *KCNE5* (potassium voltage-gated channel subfamily E regulatory subunit 5).

CASK is a cytoskeleton protein belonging to the family of membrane-associated guanylate kinase proteins. Pathogenic mutations in CASK have been identified in brain malformation, Opitz-Kaveggia syndrome, and developmental disorders^27–29^. During embryonic neuronal development, CASK translocates to the nucleus and enhances the transcriptional activity of *TBR1* (T-box brain 1)^30^. TBR1, CASK, and CINAP (CASK interacting nucleosome assembly protein) form a complex that induces transcription of genes containing TBR1 binding sequences, such as *REELIN* (RELN)^30^.

*REELIN* mRNA expression was significantly enhanced in the patients carrying the risky SNP compared to individuals carrying the wild-type allele (Fig. 3G). RELN is a glycoprotein and acts as a ligand for the receptor LRP8 (ApoER2). RELN-LRP8 signaling has been identified to inhibit GSK3β-dependent phosphorylation and subsequent degradation of β-catenin, allowing non-phosphorylated β-catenin translocation to the nucleus and activation of gene transcription^31,32^. Furthermore, β-catenin regulates the transcription factors *NR4A1* and *NR4A2* (via *TCF4*/*LEF1*) of *CYP11B2* and aldosterone synthesis, causing increased blood pressure^14^. This suggests that genetic effects on the expression of CASK may promote APA formation.

Another candidate gene from the e-QTL analysis is *KCNE5,* which has down-regulated expression. The family of KCNE proteins comprises regulatory subunits for voltage-gated potassium (Kv) channels. Among the KCNE proteins, KCNE5 has a time and voltage-dependent inhibitory effect on KCNQ1 and KCNQ4 channels^33,34^. Sequence variants in *KCNE5* have been suggested to be associated with atrial fibrillation and abnormal electrical activity of the heart (Brugada syndrome)^34^. It may be speculated that a decrease in the expression of *KCNE5* contributes to the depolarization of the membrane, opening of the voltage-gated calcium channels, and a rise in cytoplasmic calcium, thus stimulating aldosterone production.

In conclusion, our findings provide some evidence for a potential candidate gene on chromosome X that is related to APA susceptibility. However, our GWAS-eQTL exercise requires a larger number of APA patients or functional studies to substantiate the role for aldosterone production and APAs formation.

## Methods

### Samples and nucleic acid isolation

Samples of tumor tissue from 35 APAs were collected from three different centers in Norway, Sweden, and Germany. The diagnosis of PA was based on the Endocrine Society’s clinical practice guidelines^3,35^. All patients had either a pathologic ARR (> 200) or plasma aldosterone concentration (> 150 ng/l). Therefore, PA was suspected in these patients, and the patients underwent computed tomography (CT) scan, which showed a unilateral adrenal tumor.

All of these patients underwent venous catheterization, and a lateralization was observed in these cases^3^. Therefore, the patients underwent surgery. The tumors were snap frozen immediately after surgery and stored at -80 °C until analysis. The methods used for DNA and RNA extraction have been described previously^22^. DNA and RNA were available for 35 and 34 APAs, respectively. The secondary cohort consisted of 83 sporadic APA cases from Bergen, Norway, and in total, there were 118 APAs. ARR and plasma aldosterone concentrations were missing for two German cases (G5 and G6). However, the criteria were followed for them as well. DNA was isolated from a regional control population of 760 individuals, who were randomly identified from the population registry.

### Study approval

Written informed consent was obtained from patients prior to inclusion in the study. All of the studies were conducted with approval from the ethical committee of Linkoping University, Sweden (Dnr 98,110 and 2010/40–31, Linköping University, Sweden). All experimental protocols were performed according to the relevant guidelines.

### Genotyping

For the GWAS discovery stage, genome-wide genotyping was performed on 35 APAs and 60 Swedish controls using Affymetrix Gene Chip arrays (Affymetrix SNP 6.0) according to the manufacturer’s protocol. For the replication stage, the most significant SNP from the discovery stage, rs2224095 (C__15869611_10, Applied Biosystems), was genotyped on 83 additional APAs in a Norwegian cohort and 760 Swedish healthy controls using Applied Biosystems TaqMan genotyping assays.

### Statistical and bioinformatics analysis

Quality checks were performed on a per-SNP and per-sample basis. SNPs were removed if they had a success rate of < 97% or a MAF of < 1% as well as a deviation from the Hardy–Weinberg equilibrium (*P* < 1 × 10^−6^ for deviation). Samples were similarly checked for a genotyping success rate of < 95% and their heterozygosity rate (supplementary Figs. 1A and C). Principal-component analysis was used to correct for population stratification using SNPs common to cases and controls (supplementary Fig. 1B). Duplicates or probable relatives were checked based on pairwise identity by state (IBS) according to their PI-HAT value in PLINK (PI_HAT > 0.25).

After performing these quality-control measures, 33 cases and 58 controls remained for downstream analysis. In the discovery stage, we analyzed a total of 727,307 SNPs. The relationship between each variable genetic SNP marker and APA disease was measured using logistic regression with sex as a covariate. After the analysis, a few occasional SNPs on each chromosome with a significant P-value were identified. However, they appeared as singletons, and neighboring SNPs did not show any significant association. Therefore, they were excluded from further study.

### Association and Manhattan plot

The LocusZoom tool was used to generate a regional association plot with a 2000-kb region centered on the index SNP^36^. Manhattan plots were generated with R Bioconductor software (R version 3.2.2).

### Transcriptome analysis

RNA samples of 34 APAs were prepared and loaded on an Affymetrix Human Transcriptome Array 2.0 according to manufacturer’s protocol. The array data were normalized using a Robust Multi-Array Average (RMA) sketch.

### Mutation analysis

The entire coding sequence of *MAGEE1* was selected for Sanger sequencing on an ABI 3500 Genetic Analyzer using a BigDye Terminator v3.1 cycling kit for labeling of the samples. The primer sequences and PCR conditions are available in supplementary Table 2. Mutations in *KCNJ5*, *ATP1A1*, *ATP2B3,* and *CLCN2* have been published earlier, and the sequencing method has been described^13,37^. In these cases, sequencing was performed using Sanger sequencing. The mutation rate is lower than that in immunohistochemistry-guided next-generation sequencing^38^. However, for all remaining mutation-negative APAs, we have sequenced the whole exome with high coverage without finding any mutations in any of the previously known susceptibility genes. This indicates that no small clones of unidentified mutations in these genes escaped detection.

### Expression quantitative trait loci analysis (eQTL)

Genotypes of SNP rs2224095 were retrieved from GWAS for 34 APAs for which we had expression data, and eQTL analysis was performed using the Matrix eQTL R-package^21^. Sex and age were used as covariates for statistical analysis.

## Supplementary Information

Supplementary Information.
