# Supplementary material for: X-chromosome variants are associated with aldosterone producing adenomas
Source: Sci Rep. 2021 May 18;11:10562. doi: 10.1038/s41598-021-89986-8 (PMC8131628; doi:10.1038/s41598-021-89986-8)
Supplement: Supplementary file 1 — Supplementary Information. [file 41598_2021_89986_MOESM1_ESM.pdf]

X-chromosome variants are associated with aldosterone producing adenomas

Ravi Kumar Dutta[1#], Malin Larsson[2], Thomas Arnesen[3,4,5], Anette Heie[3,4], Martin Walz[6], Piero Alesina[6], Oliver Gimm[7]*, Peter Söderkvist[1]*

[1]Division of Cell Biology, Department of Biomedical and Clinical Sciences, Linköping University, 58183, Linköping, Sweden

[2]Science for Life Laboratory, Department of Physics, Chemistry and Biology, Linköping University, 58183, Linköping, Sweden

[3]Department of Surgery, Haukeland University Hospital, Bergen, Norway

[4]Department of Biomedicine, University of Bergen, Bergen, Norway

[5]Department of Biosciences, University of Bergen, Bergen, Norway

[6]Klinik für Chirurgie and Zentrum für Minimal Invasive Chirurgie, Klinikum Essen-Mitte, Essen, Germany

[7]Department of Surgery and Department of Biomedical and Clinical Sciences, Linköping University, 58183, Linköping, Sweden

*These authors contributed equally to this study.

Corresponding author

[#]Ravi Kumar Dutta

Department of Clinical and Experimental Medicine

Linköping University

SE-58183 Linköping

Sweden

Telephone: +46-739847533

Fax: +46-10-1034273

E-mail: ravi.kumar.dutta@liu.se

Conflict of interest: The authors have declared that no conflict of interest exists.

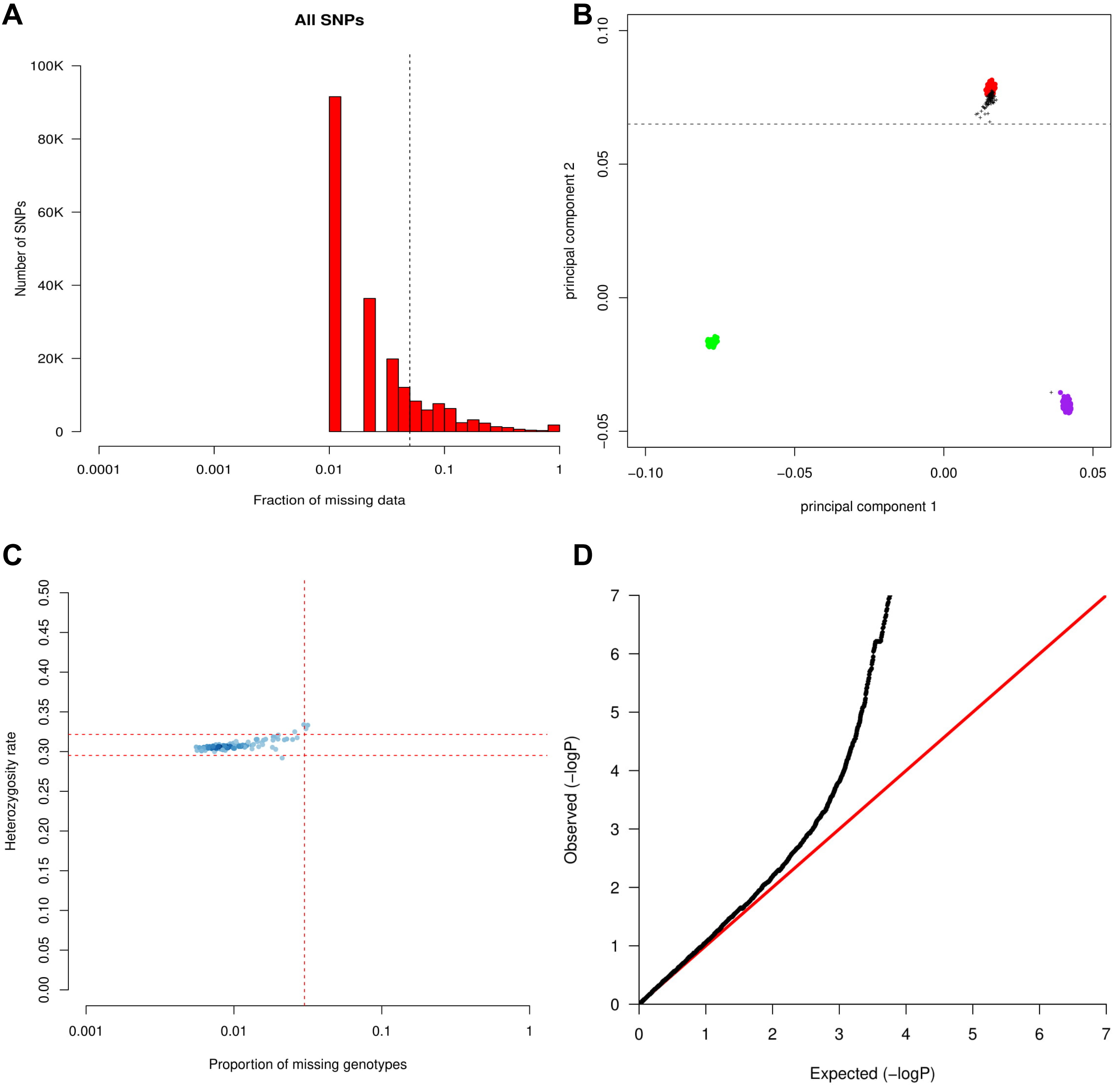

Supplementary Figure 1. (A) Histogram represents the rate of missing data across all individuals passing ‘per-individual’ quality control. **(B)** Principal-component analysis used to correct for population stratification using SNPs common to cases and controls. HapMap3 reference samples are CEU (red), CHB + JPT (purple) and YRI (green). Genome-wide association samples are shown as black crosses. Cases (n = 33) and controls (n = 58) with a second principal component score less than 0.072 (gray dashed line) were marked for removal. **(C)** Samples with excess heterozygosity rate and missing genotypes were removed (threshold-dashed lines). **(D)** Quantile-quantile plots of association in the GWA data, based on $\chi 2$ statistics from a test of allelic association. CEU -Utah residents with Northern and Western European ancestry from the CEPH collection, CHB - Han Chinese in Beijing, China, JPT -Japanese in Tokyo, Japan, YRI - Yoruba in Ibadan, Nigeria

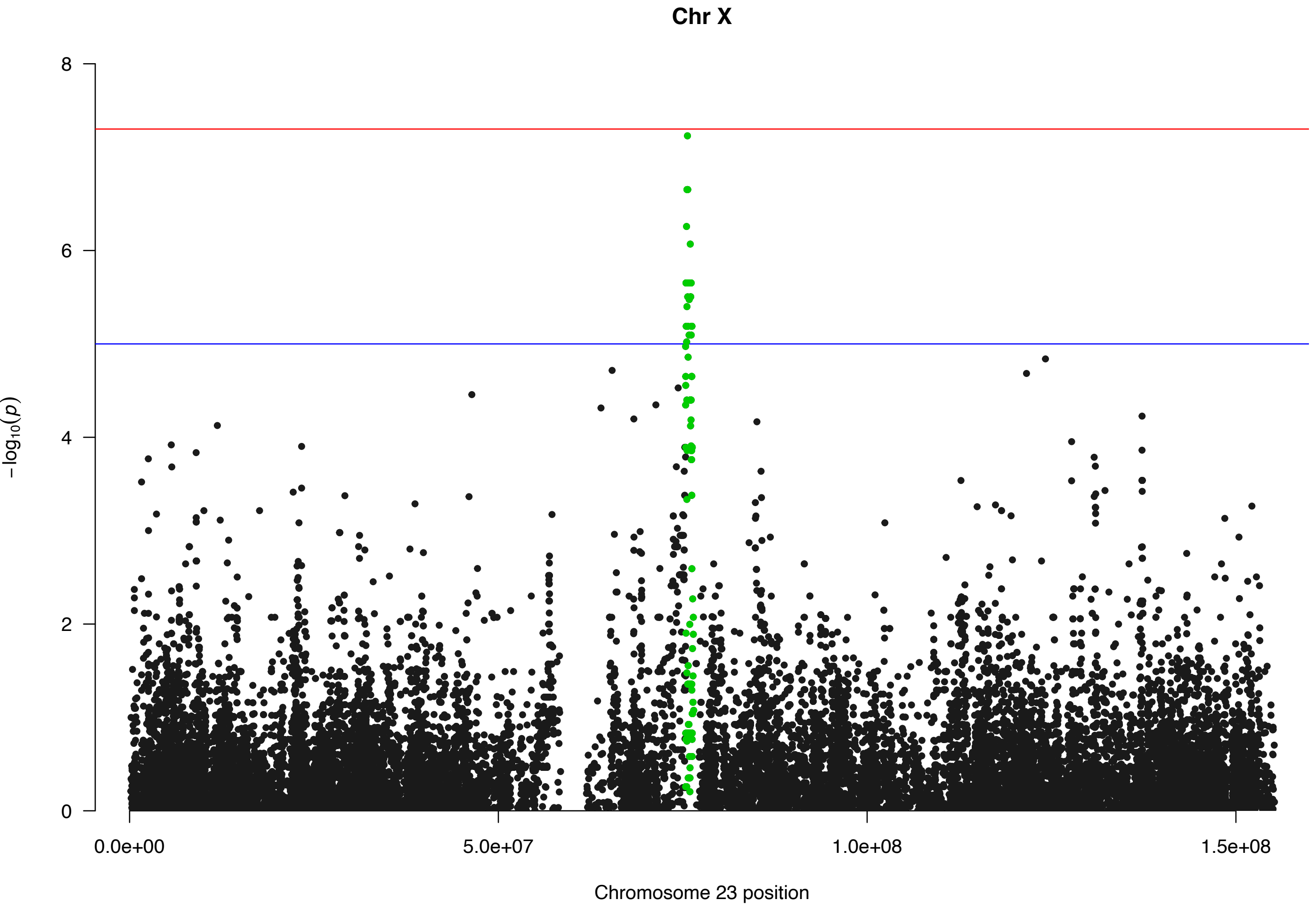

Supplementary Figure 2. The plot shows discovery association results of X-chromosome SNPs in GWAS samples. The –log10 P values (y axis) of the SNPs are shown according to their X-chromosome positions (x axis). Green dots show the 100 significant SNPs in the region of susceptibility loci.

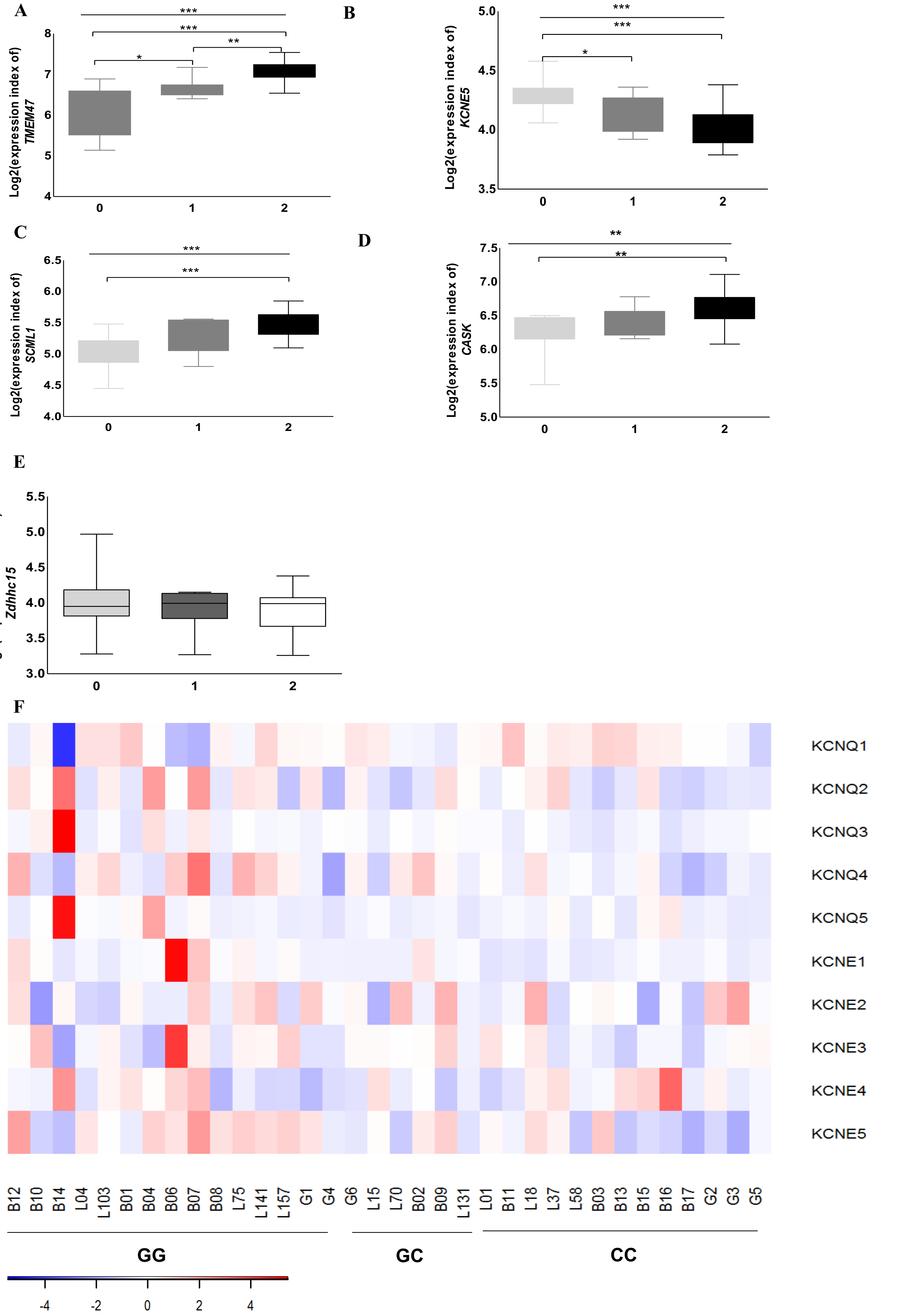

Supplementary Figure 3. **eQTL analysis. (A-F)** Log10 expression of *TMEM47*, *KCNE5*, *SCML1, CASK* and *Zdhhc15* after normalization of mRNA microarray data. *P<0.05, **P<0.005, ***P<0.0005, 2-tailed student t test and ANOVA. **(E)** Heatmap of *KCNQ1-5* and *KCNE1-5* genes (n=34). 2-tailed student t test. Genotypes 0=GG, 1=GC, 2=CC

Table S1. Clinical characteristics of the patient with aldosterone producing adenomas.

Case ID	Gender	Age (Years)	Aldosterone (ng/l)	Pre-operative Renin (ng/mL/h)	Tumor size (mm)	potassium(mmol/L)	mutated gene
L001	M	39,1	580	3	30	2,8	KCNJ5
L004	M	65,7	1640	6	10	2,4	CACNA1D
L015	F	49,1	290	1	7	3,2	ATP2B3
L018	M	57,5	320	<2	17	2,7	✷
L037	M	58	470	2	10	2,8	ATP1A1
L058	F	45,9	530	1	10	2,8	✷
L070	F	32,1	980	2	25	2,9	KCNJ5
L075	F	30,3	919	8	10	3,7	CTNNB1
L103	M	39,4	760	3	10	2,7	✷
B001	M	64,3	1246	0,2	11	NA	ATP2B3
B002	F	37,7	1675	0,1	17	NA	KCNJ5
B003	M	51,9	1599	0,4	NA	NA	✷
B004	M	58,7	624	0,6	8	NA	CACNA1D
B005	M	39,1	428	0,1	7	NA	✷
B006	M	64,6	1108	0,1	20	NA	✷
B008	M	68,3	918	0,2	NA	NA	✷
B009	F	36,4	1013	0,2	37	NA	KCNJ5
B010	F	35,3	793	0,2	12	NA	✷
B011	M	51,8	757	0,4	8	NA	✷
B012	M	50,7	643	1,2	15	NA	✷
B013	M	58,1	1564	0,5	17	NA	CACNA1D
B014	M	51,8	876	0,2	10	NA	✷
B015	M	57,9	1266	0,6	12	NA	✷
B016	M	35,5	791	0,2	13	NA	CLCN2
B017	M	47,7	1078	0,9	17	NA	KCNJ5
G1	M	54,9	300	<1.0	26	NA	KCNJ5
G2	M	60,5	350	1.4	15	NA	ATP1A1
G3	F	69,4	329	7.0	19	NA	KCNJ5
G4	M	60,9	184	0.6	15	NA	KCNJ5
G5	M	46,9	NA	NA	40	NA	✷
G6	F	59,1	NA	NA	11	NA	KCNJ5
L131	F	44,2	390	2	15	3,4	KCNJ5
L141	M	44,8	460	3	12	2,3	ATP2B3
L157	F	55,9	1140	8	25	3,7	✷

✷Tumors do not have mutations in any known susceptibility genes. NA, Data is not available.

Table S2. Sequencing primers of *MAGEE1* gene

	Forward	Reverse
1	CTGTCGGTGTCTGCTCCTAC	CAGGGAGGTGCTCGGTAC
2	CCTCTGAGGTACCGAGCA	CCCTAGGGCTTGGCAAAAC
3	TGATGGATCGGACACCTCC	GAATCATCATCAGGAGGCCC
4	AGAATATCGCAACCAGTTTCCT	GATGGTACCTCTGCAGCAG
5	GCCAGAGAAATACAACGAAGCT	TCCCAGCTTGAACCCCTAAG
6	AGTGGTACTTGATCCCAGGA	GGTCAATCACATTTACCCAATCA

MAGEE1 is single exon gene. Primers are overlapping and covers the entire exon.

Table S3. Expression quantitative trait loci (eQTL)analysis identified 24 Trans-eQTL.

**snps**	**gene**	**statistic**	**pvalue**	**FDR**	**beta**	**gene**
rs2224095	TC0X000948.hg.1	4.88823342184745	2.7e-05	0.05	0.440759493670886	TMEM47
rs2224095	TC0X000087.hg.1	3.94846480979584	0.0004	0.2	0.193924050632911	SCML1
rs2224095	TC0X000357.hg.1	3.58027820281114	0.001	0.4	0.26162447257384	HEPH
rs2224095	TC0X001072.hg.1	3.54249572854588	0.001	0.4	0.157594936708861	FAM120C
rs2224095	TC0X000175.hg.1	3.35296062435385	0.002	0.5	0.588037974683544	SYTL5
rs2224095	TC0X001541.hg.1	-3.319886499	0.002	0.5	-0.06938818565400	IKBKG
rs2224095	TC0X000528.hg.1	3.25130473874681	0.002	0.5	0.208565400843882	MID2
rs2224095	TC0X000350.hg.1	3.18064120929828	0.003	0.6	0.184831223628692	TLE1P1
rs2224095	TC0X000527.hg.1	3.13184006510742	0.003	0.6	0.103776371308017	NCBP2L
rs2224095	TC0X001353.hg.1	3.0971699761324	0.004	0.6	0.174641350210971	SMARCA1
rs2224095	TC0X000969.hg.1	3.06500882595313	0.004	0.6	0.163755274261603	CASK
rs2224095	TC0X000721.hg.1	-3.030014432947	0.004	0.6	-0.07613924050632	MAMLD1
rs2224095	TC0X000724.hg.1	2.98243619270069	0.005	0.6	0.0788818565400844	MTMR1
rs2224095	TC0X000042.hg.1	2.94363589757819	0.005	0.6	0.139662447257384	HCCS
rs2224095	TC0X000952.hg.1	2.91559383295148	0.006	0.6	0.149113924050633	DYNLT3
rs2224095	TC0X000941.hg.1	2.89611425084308	0.006	0.6	0.151054852320675	TAB3
rs2224095	TC0X001405.hg.1	2.86027838686834	0.007	0.6	0.175021097046413	ZNF75D
rs2224095	TC0X000681.hg.1	2.85924755192462	0.007	0.6	0.101392405063291	HNRNPA3P3
rs2224095	TC0X002300.hg.1	2.83833037294872	0.007	0.6	0.297616033755274	TSPAN7
rs2224095	TC0X002336.hg.1	-2.824750597552	0.008	0.6	-0.09930379746835	KCNE5
rs2224095	TC0X000648.hg.1	2.79571059641535	0.008	0.6	0.134008438818565	ZNF449
rs2224095	TC0X002308.hg.1	-2.784518476564	0.008	0.6	-0.07662447257383	ARHGAP4
rs2224095	TC0X001208.hg.1	2.76790069966879	0.009	0.6	0.1642194092827	TSPAN6
rs2224095	TC0X001312.hg.1	2.74593214905627	0.009	0.6	0.242215189873418	sep6
